# MyD88 Deficiency Alters Expression of Antimicrobial Factors in Mouse Salivary Glands

**DOI:** 10.1371/journal.pone.0113333

**Published:** 2014-11-21

**Authors:** Takeshi Into, Toshiya Takigawa, Shumpei Niida, Ken-ichiro Shibata

**Affiliations:** 1 Department of Oral Microbiology, Division of Oral Infections and Health Sciences, Asahi University School of Dentistry, Gifu, Japan; 2 Department of Oral Anatomy, Division of Oral Structure, Function and Development, Asahi University School of Dentistry, Gifu, Japan; 3 Laboratory of Genomics and Proteomics, National Center for Geriatrics and Gerontology (NCGG), Aichi, Japan; 4 Laboratory of Oral Molecular Microbiology, Department of Oral Pathobiological Science, Hokkaido University Graduate School of Dental Medicine, Hokkaido, Japan; National Institutes of Health, United States of America

## Abstract

The surfaces of oral mucosa are protected from infections by antimicrobial proteins and natural immunoglobulins that are constantly secreted in saliva, serving as principal innate immune defense in the oral cavity. MyD88 is an important adaptor protein for signal transduction downstream of Toll-like receptors and TACI, receptors for regulation of innate immunity and B cell responses, respectively. Although MyD88-mediated signaling has a regulatory role in the intestinal mucosal immunity, its specific role in the oral cavity has remained elusive. In the present study, we assessed the influence of MyD88 deficiency on the oral innate defense, particularly the expression of antimicrobial proteins in salivary glands and production of salivary basal immunoglobulins, in mice. Microarray analysis of the whole tissues of submandibular glands revealed that the expression of several genes encoding salivary antimicrobial proteins, such as secretory leukocyte peptidase inhibitor (SLPI), S100A8, and lactotransferrin, was reduced due to MyD88 deficiency. Histologically, SLPI-expressing acinar cells were evidently decreased in the glands from MyD88 deficient mice compared to wild-type mice. Flow cytometric analysis revealed that B cell populations, including B-1 cells and IgA^+^ plasma cells, residing in submandibular glands were increased by MyD88 deficiency. The level of salivary anti-phosphorylcholine IgA was elevated in MyD88 deficient mice compared to wild-type mice. Thus, this study provides a detailed description of the effect of MyD88 deficiency on expression of several salivary antimicrobial factors in mice, illustrating the role for MyD88-mediated signaling in the innate immune defense in the oral cavity.

## Introduction

Saliva, which is secreted from the salivary glands (SGs), is one of the major body fluids. The lubricative function of saliva is important for protection of the oral mucosal surfaces from desiccation, wetting foods and facilitating the initiation of swallowing. The salivary digestive enzymes are essential in the processing of dietary starches and fats. Antimicrobial agents are also contained in saliva, constantly protecting the surfaces of oral mucosa from infections. Indeed, a variety of antimicrobial proteins (AMPs), including bactericidal peptides and enzymes, and natural immunoglobulins (Igs), including IgA and IgM, are constantly secreted [1], [2], [3]. They are thought to serve as the principal innate immune defense in the oral cavity.

Toll-like receptors (TLRs) are major receptors for sensing the presence of microbes through recognition of specific molecular patterns conserved in various classes of microbes [4]. After recognition of cognate patterns, they activate signaling for induction and regulation of cellular responses associated with innate immunity [5]. MyD88 (myeloid differentiation factor 88) serves as an important signaling adaptor for TLRs [6]. In various types of cells, MyD88-mediated signaling activates the transcription factors NF-κB and AP-1, among others, ultimately leading to transcription of immune regulators, such as cytokines, and of antimicrobial agents including AMPs [5], [7]. Meanwhile, MyD88 also has a role in the control of B cell responses through mediation of signaling downstream not only of TLRs but also of TACI, a receptor for the B cell cytokines BAFF and APRIL [8], [9]. In B cells, TACI-triggered MyD88-mediated signaling induces activation of NF-κB and the expression of activation-induced cytidine deaminase for appropriate class switch recombination [9].

It has been shown that mouse MyD88 deficiency leads to susceptibility to infections of various pathogens and human MyD88 deficiency occasionally exposes patients to life-threatening pyogenic bacterial infections [5], [10], [11]. In addition, crucial defensive roles for MyD88 in the intestinal mucosal immunity have been elucidated using MyD88 deficient mice [12], [13], [14]. In the intestinal mucosal immunity, B cell-intrinsic MyD88 drives signaling for IgM production to prevent systemic dissemination of intestinal microbiota [13]. Moreover, several reports indicated that MyD88 is essential for basal production of intestinal IgA [15], [16]. In contrast, it has not been clearly elucidated whether MyD88 deficiency influences the innate immune defense in the oral cavity. In the present study, we aimed to investigate the effect of MyD88 deficiency on the innate defense in the oral cavity, particularly expression of AMPs in SGs and production of salivary Igs.

## Materials and Methods

### Mice

C57BL/6 background *Myd88*-deficient (*Myd88* ^-/-^) mice originally established in the Akira laboratory [17] were obtained from Oriental Bio Service (Kyoto, Japan). These mice were backcrossed for at least 6 generations with conventionally raised control mice (B57BL/6J Jms Slc; Japan SLC, Shizuoka, Japan) before starting this study. All of the *Myd88* ^-/-^ and wild-type (*Myd88* ^+/+^) control mice that were used in the experiments were offspring of heterozygous parents and were matched by age (10 weeks) and sex within the same experiment.

For collection of saliva, mice were anesthetized with intraperitoneal somnopentyl (35 mg/kg; Kyoritsu Seiyaku Corporation, Tokyo, Japan) and salivation was promoted by a simultaneous injection of pilocarpine hydrochloride (6 mg/kg; Tokyo Chemical Industry, Tokyo, Japan). Mice were positioned on their sides with heads pointing slightly down to facilitate saliva collection using a micropipette. For other experiments, the mice were killed by cervical dislocation and major SGs and spleens were removed.

All mice, 2 to 4 mice per cage, were maintained in the animal facility at the Asahi University School of Dentistry. Mice were fed water and a radiation-sterilized diet *ad libitum* with HEPA-filtered air in the conventional animal room (23±2°C, 50% humidity, 12 h light/dark cycle). This study was carried out in accordance with the recommendations in the Guide for the Care and Use of Laboratory Animals of the National Institutes of Health. The protocol was approved by the Committee on the Ethics of Animal Experiments of the Asahi University (Permit Number: 11-028 and 12-001). All efforts were made to minimize suffering of animals.

### Histological analysis

SGs collected from male *Myd88* ^+/+^ and *Myd88* ^-/-^ mice (10 weeks of age) were immersed in periodate-lysine-paraformaldehyde-fixative for 6 h at 4°C, embedded in paraffin and serially sectioned at 5 µm of thickness. Sections were stained with hematoxylin and eosin and histologically analyzed. For immunofluorescent staining, the tissue sections were deparaffinized and immersed in distilled water. The sections were treated with 0.1% proteinase K in phosphate-buffered saline (PBS) for 5 min at room temperature and washed three times with PBS. The sections were then blocked with 1% bovine serum albumin (BSA) in PBS followed by incubation for 1 h at room temperature with rabbit anti-SLPI (secretory leukocyte peptidase inhibitor) antibody (OAPB00538; Aviva System Biology, San Diego, CA, USA). After washing three times with PBS, the sections were incubated for 30 min at room temperature with Alexa Fluor 488-conjugated anti-rabbit IgG antibody (Life Technologies, Rockville, MD, USA) and propidium iodide. After washing with PBS, the sections were sealed in the presence of Prolong Gold anti-fade reagent (Life Technologies). Optical and fluorescent images were obtained using an SZ stereomicroscope with DP21 digital camera (Olympus, Tokyo, Japan), a BX41 microscope (Olympus) and, a Biozero fluorescence microscope (KEYENCE, Osaka, Japan) and processed using Adobe Photoshop (Adobe, San Jose, CA).

### Total RNA extraction from SGs

Major SGs were stereoscopically dissected to remove the lymph nodes and connective and fatty tissues and divided into the sublingual gland (SLG) and submandibular gland (SMG). These tissues were temporarily stored in ice-cold RNA*later* solution (Life Technologies), and then quickly frozen in liquid nitrogen and stored at −80°C. Thawed tissues were homogenized in TRIzol reagent (1 ml per 100 mg of tissue; Life Technologies) using gentleMACS M tubes in a gentleMACS Dissociator (Miltenyi Biotech, Bergisch Gladbach, Germany). Total RNA was extracted using a PureLink RNA mini kit (Life Technologies), according to the manufacturer's instructions.

### Microarray analysis of SMGs

For the linear T7-based cRNA amplification, 100 ng of total RNA extracted from SGs was used. To generate Cy3-labeled cRNA, RNA was amplified and labeled using the Agilent Low Input Quick Amp Labeling Kit (Agilent Technologies, Santa Clara, CA, USA). The hybridization procedure was performed according to the Agilent 60-mer oligo microarray processing protocol using the Agilent Gene Expression Hybridization Kit (Agilent Technologies). Briefly, 600 ng of Cy3-labeled fragmented cRNA in hybridization buffer was hybridized overnight (17 h, 65°C) to Agilent Whole Mouse Genome Oligo Microarrays (8×60K) using hybridization chamber and oven. The microarrays were washed once with the Agilent Gene Expression Wash Buffer 1 for 1 min at room temperature followed by a second wash with preheated Agilent Gene Expression Wash Buffer 2 (37°C) for 1 min. The last washing step was performed with acetonitrile. Cy3 fluorescence signals of the hybridized Agilent Microarrays were detected using Agilent's Microarray Scanner System. Data were analyzed using Agilent Feature Extraction Software and Rosetta Resolverâ gene expression data analysis system (Rosetta Biosoftware, Seattle, WA, USA). The dataset of microarray is available from the Gene Expression Omnibus (accession number, GSE61339).

### Quantitative reverse transcription-polymerase chain reaction (qRT-PCR)

Total RNA (1 µg) was reverse-transcribed using ReverTra Ace qPCR RT master mix with gDNA remover kit (TOYOBO, Osaka, Japan) with oligo21dT and random hexamer primers. SYBR Green-based qRT-PCR was performed using SsoFast EvaGreen Supermix (Bio-Rad, Hercules, CA, USA) and the Thermal Cycler Dice Real-Time System TP800 (TaKaRa, Shiga, Japan). The primer set for mouse *S100a8* for SYBR Green-based qRT-PCR was obtained from QIAGEN (Hilden, Germany). All other primer sets for SYBR Green-based qRT-PCR were obtained from TaKaRa. The sequences of primers used in this study are listed in Table S1. The assessment of gene expression was determined by the ΔΔ*C_t_* method. Results shown as relative expression were normalized to levels of the housekeeping gene *Hprt1*.

### Flow cytometry

SMGs or spleens were homogenized in gentleMACS C tubes (Miltenyi Biotech) in a gentleMACS Dissociator followed by treatment with collagenase D (2 mg/ml) and DNaseI (40 U/ml) for 30 min at 37°C at 12 rpm on a rotating platform (MACSmix rotator; Miltenyi Biotech). Cells were further homogenized and filtered through 70 µm nylon cell strainers (BD Falcon, Franklin Lakes, NJ, USA), washed with PBS containing 2 mM EDTA and 0.5% BSA (PEB) and suspended in PEB at 1×10^7^ cells/ml. Then 1×10^6^ cells were treated with mouse FcR Blocking Reagent (Miltenyi Biotech) for 10 min at 4°C followed by staining with fluorescence-labeled antibodies for 20 min at 4°C. Cells were then washed with PEB, suspended in 1% paraformaldehyde in PBS and stored at 4°C. Diluted samples were analyzed by flow cytometry using an EC800 Cell Analyzer (SONY, Tokyo, Japan) and accompanying software.

FITC-labeled anti-mouse CD45, PerCP-labeled anti-mouse/human B220/CD45R, PE/Cy7-labeled anti-mouse CD138 (Syndecan-1), biotin-conjugated anti-mouse IgA and FITC-labeled anti-mouse IgM were obtained from BioLegend (San Diego, CA, USA). PE/Cy7-labeled anti-mouse CD23 and PE-labeled anti-mouse IgD were obtained from iCyt (Champaign, IL, USA). PE-labeled anti-mouse CD5 and PerCP-labeled anti-biotin were from Miltenyi Biotech. Fluorescence-labeled isotype-matched control antibodies were obtained from BioLegend.

### Measurement of Ig levels by enzyme-linked immunosorbent assay (ELISA)

Collected saliva (25 µl) was diluted 5 times with ice-cold PBS containing cOmplete Mini Proteinase Inhibitor Tablet (Roche Diagnostics, Basel, Switzerland). Whole blood was collected and incubated at 37°C to obtain sera. Fresh fecal pellets were collected and immediately weighed. Pellets were homogenized in ice-cold PBS containing protenase inhibitors (100 µl per 1 mg of fecal pellet) using gentleMACS M tubes and a gentleMACS Dissociator and subsequent clarification by centrifugation at 3,000 rpm for 5 min at 4°C to obtain fecal extracts. Sera and fecal extracts were diluted 1,000- and 10-fold, respectively, with PBS containing protease inhibitors. The levels of IgA, IgM, IgG1, IgG2c and IgG3 antibodies in diluted samples were determined using Mouse ELISA Quantitation Sets (Bethyl Laboratories, Montgomery, TX, USA) and Nunc Immunoplate Maxisorp microplates (Thermo Fisher Scientific, Waltham, MA, USA). The levels of IgA and IgM antibodies against phosphorylcholine (PC) were detected in the microplates coated with 50 µg/ml of PC conjugated with BSA (PC-1011H; Biosearch Technologies, Petaluma, CA, USA) in carbonate-bicarbonate buffer (pH 9.4) using the secondary antibodies in the ELISA kits. Unconjugated BSA was used as a negative control for ELISA. Tetramethylbenzidine (KPL, Gaithersburg, MD, USA) was used as the substrate in the reaction for color development, which was terminated by 1 N H_2_SO_4_ and measured by absorbance at 450 nm with an xMark Microplate Absorbance Spectrophotometer (Bio-Rad).

### Statistical analysis

In several experiments, data are expressed as mean ± standard deviation (SD). *P* values were calculated by unpaired Student's *t*-test and those less than 0.05 or 0.01 were considered significant.

## Results

### MyD88 deficiency affects histology of SGs

MyD88 deficiency causes shifting of the composition of the intestinal commensal microbiota [14], [18]. In isolated mouse colonies, long-term breeding of MyD88-deficient mice results in the accumulation of anomalies in microbiota [19], which may cause unexpected influence through dysbiosis. In this study, we minimized such influence of MyD88 deficiency by the restrictive use of *Myd88* ^-/-^ and *Myd88* ^+/+^ homozygous offspring of heterozygous mice that had inherited normal commensal microbiota from *Myd88* ^+/+^ mothers.

**Figure 1:**
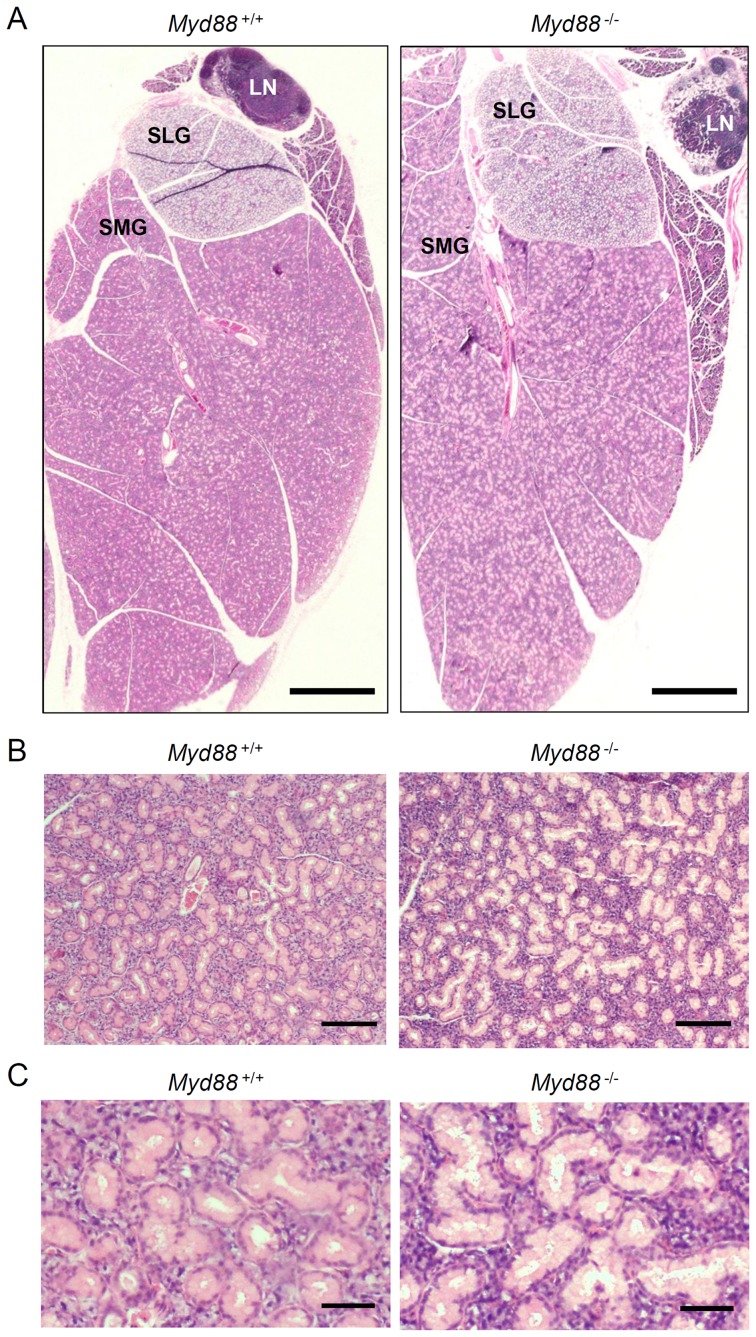
Effect of MyD88 deficiency on histology of SGs. Paraffin sections of the major SGs from male *Myd88* ^+/+^ mice (left) and *Myd88* ^-/-^ mice (right) at 10 weeks old were stained with hematoxylin and eosin. Results are representative of more than three independent experiments. A: Low magnification images of the whole tissues of major SGs. LN, lymph node, SLG, sublingual gland; SMG, submandibular gland. Scale bar, 1 mm. B: Middle magnification images of SMGs. Scale bar, 100 µm. C: High magnification images of SMGs. Scale bar, 20 µm.

To determine the effect of MyD88 deficiency on the oral innate defense, we focused on the major SGs because they are major sources of various salivary antimicrobial products. We initially evaluated the histological appearance of SGs collected from naive *Myd88* ^-/-^ mice as well as *Myd88* ^+/+^ control mice. MyD88 deficiency barely affected morphological characteristics of the whole tissues (Figure 1A), but was found to affect the histology of SMGs. Compared to *Myd88* ^+/+^ mice, the lumina of intercalated ducts that are conspicuously found in mouse SMGs were slightly enlarged probably due to decrease of cellular height in duct cells in *Myd88* ^-/-^ mice, while the number of ducts was essentially unchanged (Figure 1B, 1C). Additionally, cells were considerably congested throughout the region of acini in SMGs from *Myd88* ^-/-^ mice (Figure 1C). Evident pathological states, such as inflammation and infections, were not observed.

To evaluate whether such histological changes affect the function of SGs, we tested pilocarpine-stimulated salivation. As a result, secretion of saliva was almost identical in *Myd88* ^-/-^ mice and *Myd88* ^+/+^ mice (data not shown).

### MyD88 deficiency reduces expression of SLPI in SGs

**Figure 2:**
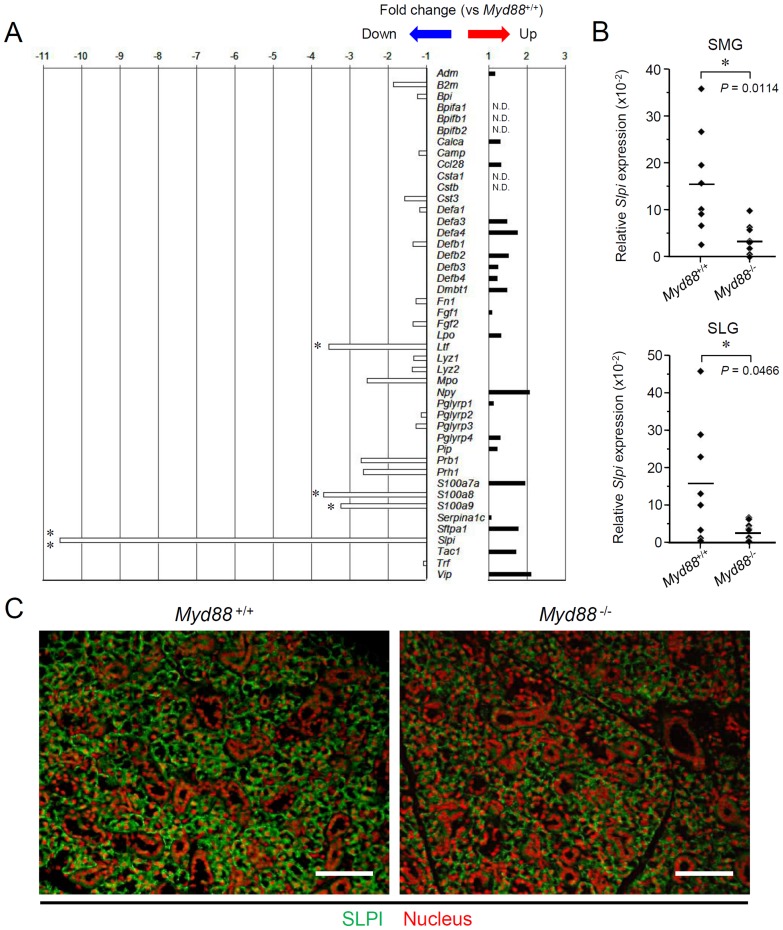
Analysis of expression of the genes encoding salivary AMPs in SGs. A: Microarray analysis of the expression of 45 genes encoding salivary AMPs. Total RNA was prepared from SMGs from *Myd88* ^+/+^ mice and *Myd88* ^-/-^ mice at 10 weeks old (n = 3 each). The mean value of fold-change for each gene in *Myd88* ^-/-^ mice shown here was calculated relative to the mean value of expression of that gene in *Myd88* ^+/+^ mice. Statistical analysis was performed by unpaired Student's *t*-test. The complete list of 45 genes is in Table S2. *, *P*<0.05, **, *P*<0.01, N.D., not detectable. B: qRT-PCR analysis of *Slpi* expression. Total RNA was prepared separately from SMGs and SLGs from *Myd88* ^+/+^ mice and *Myd88* ^-/-^ mice at 10 weeks old (n = 8 each). Each expression level of *Slpi* was calculated relative to expression of the *Hprt1* housekeeping gene. Means of each group were shown and *P* values were calculated by unpaired Student's *t*-test. *, *P*<0.05. C: Fluorescent imaging of SLPI expression in SMGs. Sections of SMGs from male *Myd88* ^+/+^ mice (left) and *Myd88* ^-/-^ mice (right) at 10 weeks old were deparaffinized and incubated with an anti-SLPI antibody/Alexa Fluor 488-conjugated secondary antibody (green) and propidium iodide to stain nuclei (red). Results are representative of three independent experiments. Scale, 50 µm.

We next investigated expression of the genes encoding AMPs in SGs. To conveniently assess it, we performed microarrays of whole SMGs from *Myd88* ^-/-^ mice as well as *Myd88* ^+/+^ controls. The selected 45 genes (Figure 2A; and listed in Table S2) are analogous to the human genes encoding salivary AMPs [20]. The data revealed notable downregulation of *Slpi* in SMGs from *Myd88* ^-/-^ mice compared to *Myd88* ^+/+^ controls (Figure 2A). Statistically significant reduction of *Slpi* expression could be confirmed both in SMGs and SLGs from *Myd88* ^-/-^ mice by qRT-PCR (Figure 2B).

We also histologically examined the expression of SLPI protein in SMGs. Immunofluorescent staining of SLPI revealed that SLPI is mainly produced by acinar cells and is not clearly found in intercalated ducts and excretory ducts in SMGs from *Myd88* ^+/+^ mice (Figure 2C). In SMGs from *Myd88* ^-/-^ mice, the number of cells was considerably increased throughout the acinar region, but the number of SLPI producing acinar cells was evidently decreased as compared with *Myd88* ^+/+^ mice.

### Effect of MyD88 deficiency on expression of other AMPs in SGs

Based on the microarray data, the 45 genes encoding AMPs include several genes that correspond to the members of the AMP families of α-defensins, β-defensins and S100 calcium-binding proteins. These groups consist of analogous (or paralogous) genes and functionally-associated genes. Notably, expression of certain members of α-defensins, β-defensins and S100 proteins can be intrinsically induced by TLR signaling [21], [22], [23]. Therefore, it is strongly possible that MyD88 deficiency affects expression levels of the members of these AMP families. We evaluated the microarray data for expression of the detectable members of them.

**Figure 3:**
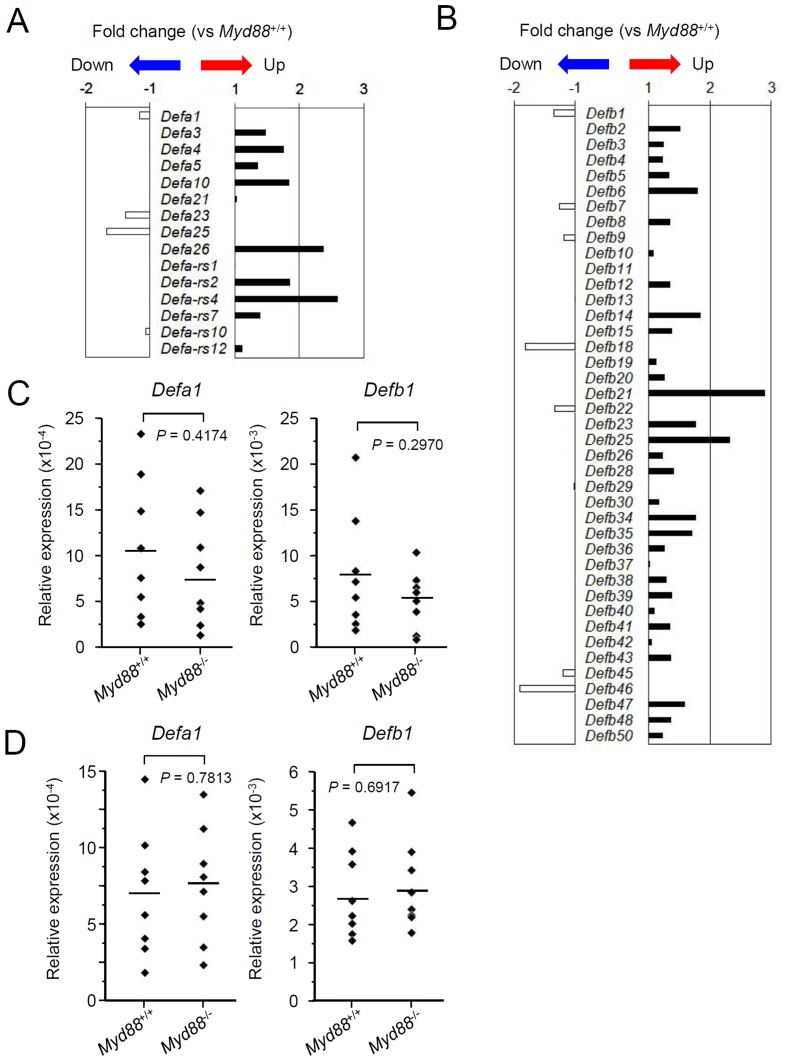
Analysis of expression of the genes encoding defensins in SGs. A and B: Microarray analysis of the genes encoding α-defensins (A) and β-defensins (B). Total RNA was prepared from SMGs from *Myd88* ^+/+^ mice and *Myd88* ^-/-^ mice at 10 weeks old (n = 3 each). The mean value of fold-change for each gene in SMGs from *Myd88* ^-/-^ mice shown here was calculated relative to the mean value of expression of that gene in SMGs from *Myd88* ^+/+^ mice. Statistical analysis was performed by unpaired Student's *t*-test. C and D: qRT-PCR analysis for expression of *Defa1* (left) and *Defb1* (right) in SMGs (C) and SLGs (D). Total RNA was prepared separately from SMGs and SLGs collected from *Myd88* ^+/+^ mice and *Myd88* ^-/-^ mice at 10 weeks old (n = 8 each). Expression levels of *Defa1 and Defb1* in SMGs and SLGs were calculated relative to expression of the *Hprt1* housekeeping gene. Means of each group were shown and *P* values were calculated by unpaired Student's *t*-test.

Expression of the genes for both α- and β-defensins was not clearly changed in *Myd88* ^-/-^ mice compared to the *Myd88* ^+/+^ controls in the data of microarray (Figure 3A, 3B). By qRT-PCR, compared to *Myd88* ^+/+^ mice, expression of *Defa1* and *Defb1* was not significantly different in SMGs and SLGs from *Myd88* ^-/-^ mice (Figure 3C, 3D). These results suggests that TLR-mediated MyD88 signaling is dispensable for basal production of defensins in SGs.

**Figure 4:**
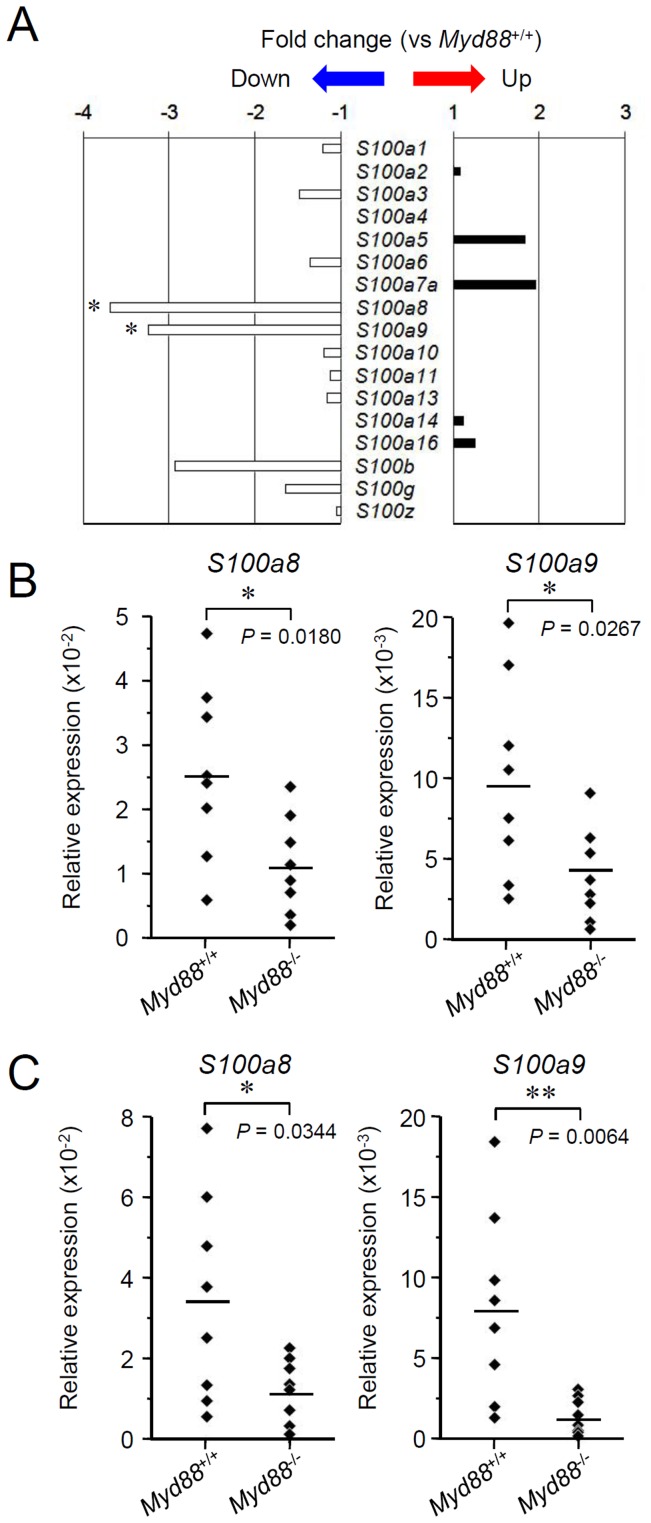
Analysis of expression of the genes encoding S100 proteins in SGs. A: Microarray analysis of the genes encoding S100 proteins. Total RNA was prepared from SMGs from *Myd88* ^+/+^ mice and *Myd88* ^-/-^ mice at 10 weeks old (n = 3 each). The mean value of fold-change for each gene in SMGs from *Myd88* ^-/-^ mice shown here was calculated relative to expression of that gene in SMGs from *Myd88* ^+/+^ mice. Statistical analysis was performed by unpaired Student's *t*-test. *, *P*<0.05. B and C: qRT-PCR analysis for expression of *S100a8* (left) and *S100a9* (right) in SMGs (B) and SLGs (C). Total RNA was prepared separately from SMGs and SLGs from *Myd88* ^+/+^ mice and *Myd88* ^-/-^ mice at 10 weeks old (n = 8 each). Expression levels of *S100a8 and S100a9* in SMGs and SLGs were calculated relative to expression of the *Hprt1* housekeeping gene. Means of each group were shown and *P* values were calculated by unpaired Student's *t*-test. *, *P*<0.05; **, *P*<0.01.

In the data of microarray, among S100 proteins, expression of *S100a8* and *S100a9* was significantly downregulated in *Myd88* ^-/-^ mice, but expression of other members was not affected (Figure 4A). By qRT-PCR, statistically significant reduction in expression of *S100a8 and S100a9* in both SMGs and SLGs from *Myd88* ^-/-^ mice was confirmed (Figure 4B, 4C), indicating that MyD88 is important for basal production of several members of the S100 protein family in SGs.

The microarray data also showed that expression of *Ltf* significantly reduced in *Myd88* ^-/-^ mice (Figure 2A). Statistically significant reduction of *Ltf* expression could be confirmed in both SMGs and SLGs from *Myd88* ^-/-^ mice by qRT-PCR (Figure S1).

### MyD88 deficiency increases B cells and IgA^+^ plasma cells in SGs

**Figure 5:**
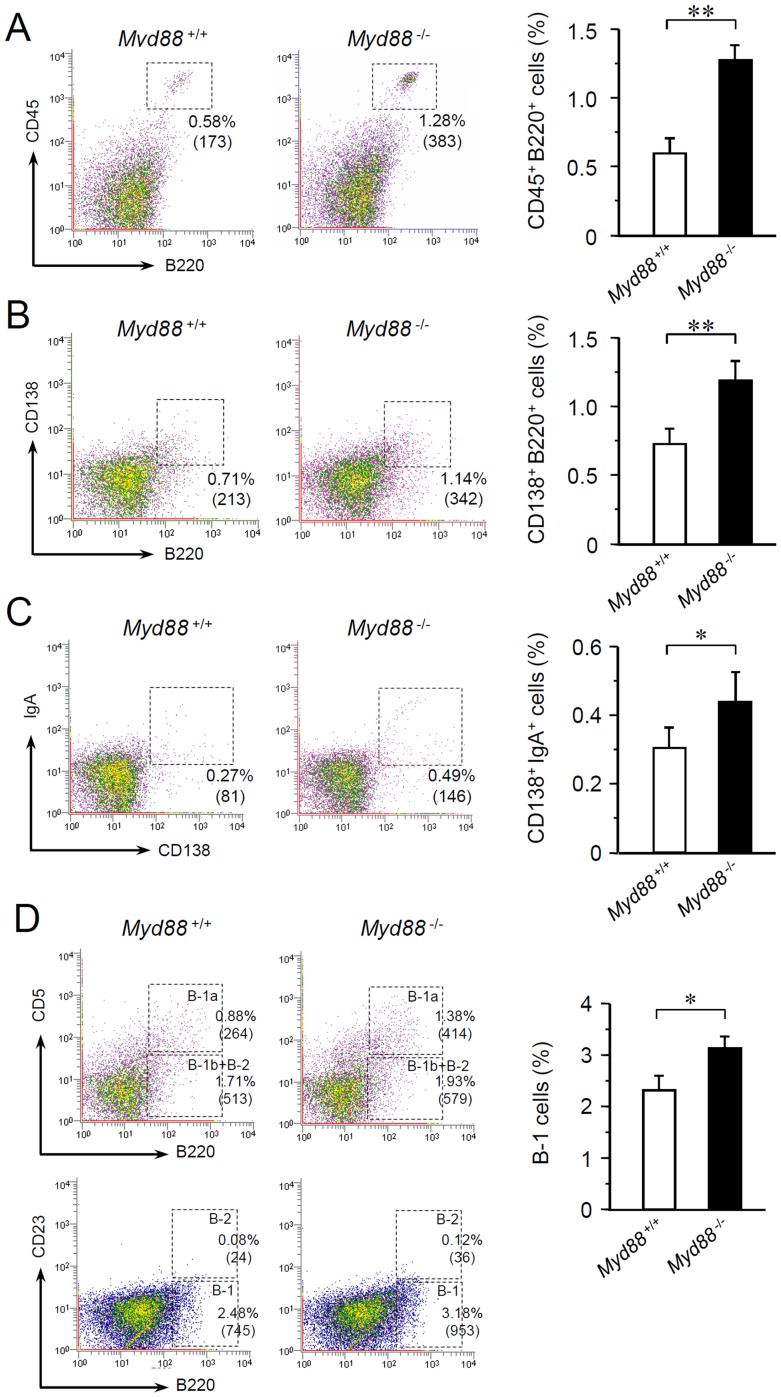
Flow cytometric analysis of B cell populations resident in SMGs. Flow cytometry was performed on cells prepared from SMGs from male *Myd88* ^+/+^ mice and *Myd88* ^-/-^ mice at 10 weeks old. A: Analysis of SMG cells obtained from four mice for expression CD45 and B220 (30,000 cells each). The percentage and cell number of the cells within the outlined area in dot plots (indicative of CD45^+^B220^+^ cells; others shown in Figure S2A) were shown. The data of percentage were graphically shown as means ± SD (n = 4 per group) and are representative of three independent experiments. **, *P*<0.01 (unpaired Student's *t*-test). B: Analysis of SMG cells obtained from four mice for expression CD138 and B220 (30,000 cells each). The percentage and cell number of the cells within the outlined area in dot plots (indicative of CD138^+^B220^+^ cells; others shown in Figure S2B) were shown. The data of percentage were graphically shown as means ± SD (n = 4 per group) and are representative of three independent experiments. **, *P*<0.01 (unpaired Student's *t*-test). **, *P*<0.01 (unpaired Student's *t*-test). C: Analysis of SMG cells obtained from four mice for expression CD138 and IgA (30,000 cells each). The percentage and cell number of the cells within the outlined area in dot plots (indicative of CD138^+^IgA^+^ cells; others not shown) were shown. The data of percentage were graphically shown as means ± SD (n = 4 per group) and are representative of three independent experiments. *, *P*<0.05 (unpaired Student's *t*-test). D: Analysis of B-1 cells and B-2 cells in SMG cells obtained from four mice (30,000 cells each). For discrimination of B-1a cells in the dot plots, B220-positive cells were separated into CD5^+^ cells (B-1a cells) and CD5^-^ cells (B-1b plus B-2 cells). For discrimination of B-2 cells, B220-positive cells were separated into CD23^+^ cells (B-2 cells) and CD23^-^ cells (B-1 cells). The percentage and cell number within the outlined area in dot plots were shown. The data of percentage of B220^+^CD23^-^ B-1 cells were graphically shown as means ± SD (n = 4 per group) and are representative of three independent experiments. *, *P*<0.05 (unpaired Student's *t*-test).

Most of the IgA found in the oral cavity is produced by IgA^+^ plasma cells residing in the major and minor SGs [2]. In addition, SMGs are known as major sources of IgA production [2]. To assess whether MyD88 deficiency affects IgA^+^ plasma cells in SMGs, we performed flow cytometric analysis of cell suspensions prepared from SMGs collected from naive *Myd88* ^-/-^ mice and *Myd88* ^+/+^ controls. SMG cells included CD45^hi^ leukocytes, most of which were revealed as B220^+^ cells indicative of B cell populations (Figure 5A; also see Figure S2A, Figure S3A). B220^+^ SMG cells included CD138^+^ plasma cells (Figure 5B; also see Figure S2B, Figure S3B). Compared to *Myd88* ^+/+^ controls, the numbers of B220^+^ cells (Figure 5A; also see Figure S2A, Figure S3A) and CD138^+^ cells (Figure 5B; also see Figure S2B, Figure S3B) were significantly elevated in SMGs from *Myd88* ^-/-^ mice. In addition, IgA^+^CD138^+^ plasma cells in SMGs were also significantly increased in *Myd88* ^-/-^ mice (Figure 5C; also see Figure S3C). Therefore, given that SGs are efficient producers of IgA, MyD88 deficiency likely increases both B cell infiltration into SGs and their differentiation into IgA^+^ plasma cells.

To investigate whether the increase in B cells or IgA^+^ plasma cells in SMGs from *Myd88* ^-/-^ mice is organ-specific or systemic, we next performed flow cytometric analysis of spleen cells. In the spleen, the majority of CD45^hi^ cells were B220^+^ cell populations (Figure S4A). The number of these B cells was elevated in *Myd88* ^-/-^ mice compared to the control, consistent with a previous finding [8]. Most of the B cells (CD138^+^ cells) that intermediately express IgM and IgD were increased in *Myd88* ^-/-^ mice (Figure S4B, histograms of IgM and IgD). On the other hand, although splenocytes include a small population of IgA^+^CD138^+^ plasma cells (or plasmablasts), their quantities were identical in *Myd88* ^-/-^ and *Myd88* ^+/+^ mice (Figure S4B, histogram of IgA).

Thus, these results collectively suggest that MyD88 deficiency causes systemic elevation of B cell development and organ-specific elevation of IgA^+^ plasma cell differentiation in SGs.

### MyD88 deficiency increases infiltration of B-1 type B cells in SGs

B cells are divided into two developmentally distinct lineages, B-1 and B-2 [24], [25]. The B-1 lineage includes two subsets termed B-1a and B-1b. We asked whether MyD88 deficiency affects the composition of B cells in SMGs. B-1a, B-1b and B-2 cells can be separated using popular markers: B220, CD5, and CD23 [26]. Flow cytometric analysis of cell suspensions prepared from SMGs revealed that both B220^+^CD5^+^ (B-1a) and B220^+^CD5^-^ (B-1b plus B-2) were increased in *Myd88* ^-/-^ mice compared with *Myd88* ^+/+^ mice (Figure 5D, upper). In addition, B220^+^CD23^-^ cells (B-1a plus B-1b) were increased in SMGs from *Myd88* ^-/-^ mice, but B220^+^CD23^+^ cells (B-2) were identical (Figure 5D, lower), indicating that MyD88 deficiency mainly elevates infiltration of B-1 type B cells into SGs.

### MyD88 deficiency affects the levels of salivary basal Igs

**Figure 6:**
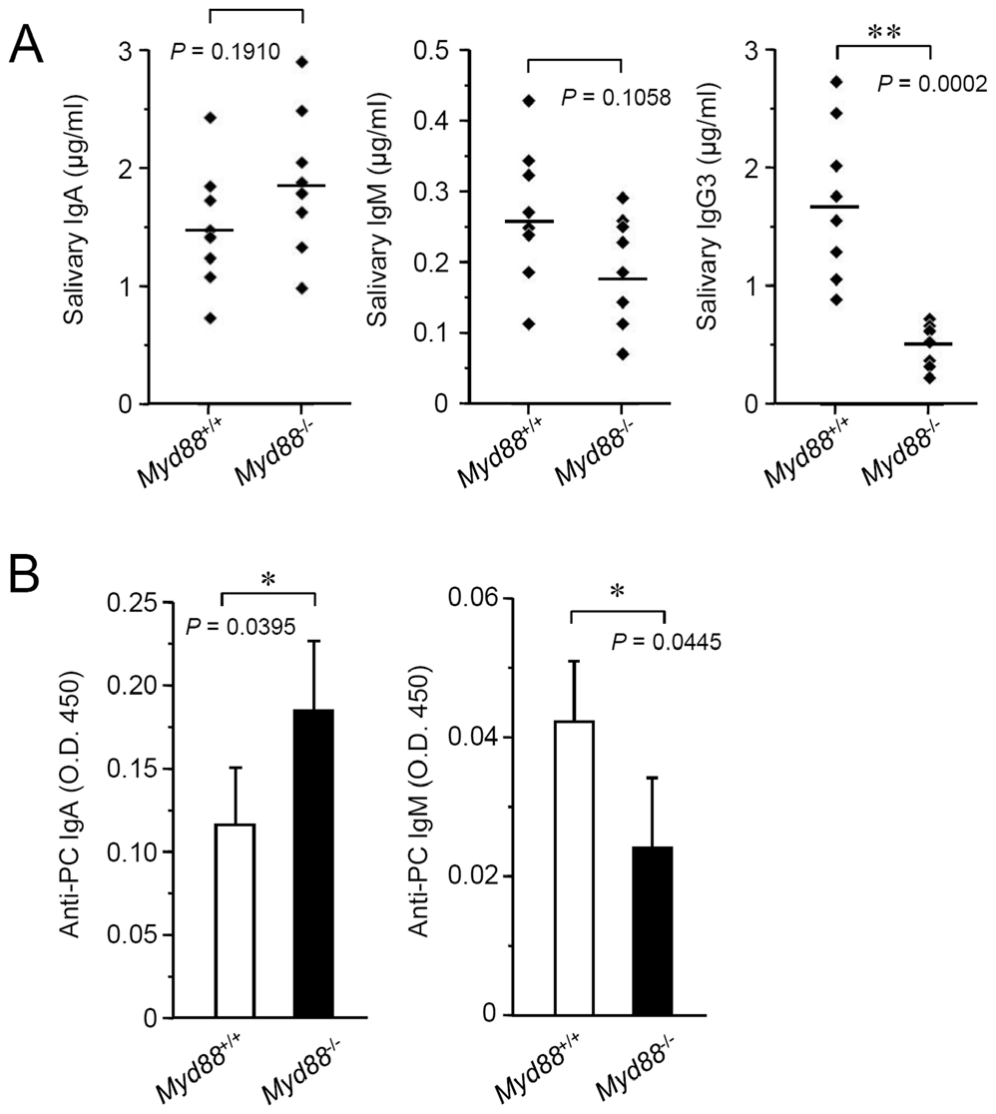
Effect of MyD88 deficiency on salivary basal Ig production. A: Levels of salivary basal IgA (left), IgM (middle) and IgG3 (right). Saliva samples were collected from naive *Myd88* ^+/+^ mice and *Myd88* ^-/-^ mice at 10 weeks old (n = 8 each) for determination of basal Ig levels by ELISA. Means of each group were shown and *P* values were calculated by unpaired Student's *t*-test. **, *P*<0.01. B: Levels of salivary anti-PC IgA (left) and anti-PC IgM (right). Saliva samples were collected from naive *Myd88* ^+/+^ mice and *Myd88* ^-/-^ mice at 10 weeks old for determination of the levels of anti-PC Igs by ELISA. Data are expressed as means ± SD (n = 4 each) and are representative of three independent experiments. *, *P*<0.05 (unpaired Student's *t*-test).

To assess whether MyD88 deficiency leads to altered salivary Ig production, we measured the levels of basal Igs in saliva collected from naive *Myd88* ^-/-^ mice as well as *Myd88* ^+/+^ controls. Compared to the controls, the levels of salivary IgA and IgM seemed slightly increased and decreased, respectively, in *Myd88* ^-/-^ mice, but they were not statistically significant (Figure 6A). In contrast to them, the level IgG3 was significantly decreased in *Myd88* ^-/-^ mice (Figure 6A). In the majority of saliva samples, the levels of IgG1 and IgG2 subclasses were not detectable (data not shown).

Most of salivary IgA is secreted from SGs, whereas salivary IgM and IgG originate from gingival crevicular fluids that are derived from plasma [2]. We determined the basal Ig levels in serum and compared them with the results of salivary Ig levels. The levels of serum IgM, IgG2c and IgG3 were reduced in *Myd88* ^-/-^ mice but the levels of IgA and IgG1 were not significantly changed as compared to the controls (Figure S5). These results at least suggest that the reduced level of salivary IgG3 in *Myd88* ^-/-^ mice is affected by its reduced level in plasma.

In the intestinal mucosal immunity, production of basal IgA is thought to be critically controlled by MyD88 signaling. Indeed, naive *Myd88*-/- mice do not produce IgA in the gut [15], [16]. However, a recent report indicated that MyD88 deficiency does not affect IgA production and it rather affects production of IgM [13]. Thus, the effect of MyD88 deficiency on the intestinal IgA production has been controversial. We therefore measured the level of intestinal IgA in the extracts obtained from fecal pellets collected from naive *Myd88* ^-/-^ mice and *Myd88* ^+/+^ controls with a method equivalent to that used in the previous report [15]. Fecal IgA was detectable in all of the samples collected from both groups of mice. The level in *Myd88* ^-/-^ mice was almost identical or slightly elevated, as compared to the controls, similarly to that of salivary IgA (Figure S6). Thus, MyD88 is dispensable for production of salivary or intestinal basal IgA.

B-1 type B cells are thought to be involved in T cell-independent production of low-affinity natural IgA and IgM [25], [27], which include broadly reactive antibodies to bacterial PC, bacterial lipopolysaccharides, double-stranded DNA and viruses [24], [28]. Such a type of Igs is thought to contribute to the innate immune defense. In addition, T cell-independent B cell responses are essentially controlled by signaling of TLRs and TACI, activation of which require MyD88 [8], [9]. We therefore examined whether MyD88 deficiency affects the level of salivary anti-PC IgA and IgM antibodies. Compared to the controls, salivary anti-PC IgA was elevated and salivary anti-PC IgM was reduced in *Myd88* ^-/-^ mice (Figure 6B).

Our results collectively suggest that increased B-1 cells in SGs of *Myd88* ^-/-^ mice produce an elevated level of salivary natural IgA, including anti-PC IgA, through a process that can be suppressed by MyD88-mediated signaling. In contrast, salivary [deleted ‘IgM and’] IgG3 that probably originate from plasma are systemically produced through a process associated with MyD88-mediated signaling.

## Discussion

This study was carried out to ascertain the effect of MyD88 deficiency on the oral innate defense. We found that MyD88 deficiency alters expression of several genes encoding AMPs in SGs. In particular, expression of the genes encoding SLPI, the S100 calcium-binding proteins S100A8 and S100A9 and lactotransferrin (also called lactoferrin) were markedly reduced. Next, MyD88 deficiency causes an increase in the number of SMG resident B cell populations, including B-1 cells and IgA^+^ plasma cells, and elevation of the level of salivary anti-PC IgA. In addition, MyD88 deficiency causes a decrease in salivary IgG3, likely indirectly, through a decrease in its plasma level. We described a detailed influence of MyD88 deficiency on salivary antimicrobial products, illustrating a potential role for MyD88-mediated signaling in the innate immune defense in the oral cavity.

MyD88 deficiency alters the histology of SMGs (Figure 1), but such alteration does not cause dysfunction of these glands. Possibly associated with the histology, expression of several genes encoding AMPs, including *Slpi*, were reduced (Figure 2). SLPI is a secreted antileukoprotease that protects epithelial tissues from various proteases, including cathepsin G, leukocyte elastase, trypsin and chymase [29]. This protein is secreted from a large variety of cell types, having been found in various body fluids, especially in mucosal fluids such as bronchial fluid and saliva. In addition to an antiprotease function, SLPI alternatively possesses antimicrobial, anti-inflammatory, and immunomodulatory functions [30]. Interestingly, in B cells, SLPI can effectively suppress class switch recombination for IgA production [31]. It is therefore possible that the elevated level of salivary anti-PC IgA in *Myd88* ^-/-^ mice is associated with the reduced expression of SLPI in SGs. It will be necessary to test whether SLPI deficiency leads to an increase in salivary IgA. It should also be investigated whether the MyD88 deficiency-associated reduction in SLPI and other antimicrobial proteins, including S100 proteins or lactoferrin, actually affects susceptibility to oral infections.

We observed increased B cells and IgA^+^ plasma cells in SGs with MyD88 deficiency and found that the majority of them were B-1 type B cells (Figure 5). In mice, B-1 cells are predominant in fetal immunity, and, in adults, they maintain the capacity to self-renew and are present primarily in serous cavities [24]. Propagated B-1 cells distribute to unusual immune sites, including the liver, spleen (red pulp), lung, uterus, and intestine. SGs are also known to contain B-1 cells, especially B-1a cells [32]. We observed that MyD88 deficiency increases both B-1a and B-1b cells in SMGs. B-1 cells produce the majority of natural IgM in the systemic immunity [24], whereas they are preferentially class-switched to produce IgA in the intestinal mucosa [27], [33]. IgA-producing B-1 cells in the intestinal lamina propria are thought to develop through processes that depend on IL-5 production [34] and existence of commensal bacteria [35]. IgA class switching of B-1 cells has also been observed in SMGs and IgA-committed B cells are thought to be derived from the nasopharynx-associated lymphoid tissues [36]. Therefore, it is possible that infiltrated B-1 cells in SMGs of *Myd88* ^-/-^ mice are directly involved in the increased production of salivary anti-PC IgA.

Salivary IgA is produced as secretory IgA in SGs through various processes, including synthesis as dimeric IgA by plasma cells that exist around secretory acini or intra-lobular ducts and transportation to the lumen by polymeric Ig receptor [2]. On the other hand, most IgG and IgM in saliva are derived mainly from plasma via the gingival crevices. Thus, salivary Igs can be affected by both oral mucosal immunity and systemic immunity. In systemic immunity, MyD88 has a role in TLR-mediated regulation of B cell responses and T cell-dependent production of IgM and IgG subclasses [8], [37]. Furthermore, MyD88 deficiency causes altered production of serum basal antibodies, including a decrease in IgM and IgG2c, probably due to a defect in B cell-intrinsic MyD88-dependent signaling [8], [13]. These observations are consistent with our data concerning their levels in serum and saliva. Conversely, inconsistent with previous findings on the intestinal IgA production [15], [16], MyD88 was not required for production of salivary and intestinal IgA. Our results suggest that MyD88 deficiency rather exerted an upregulatory effect on the processes responsible for IgA production. There is supporting evidence indicating that MyD88 deficiency leads to enhanced trafficking of intestinal bacterial antigens by CX_3_CR1^hi^ mononuclear phagocytes to the mesenteric lymph nodes, increasing IgA production in the intestinal mucosa [38]. Such antigen trafficking seems to be suppressed by recognition of intestinal microbiota by TLRs. Therefore, it is possible that MyD88-mediated signaling upon TLR recognition of oral commensal microbes influences salivary IgA production in SGs.

In conclusion, this study provides a detailed description of the effects of MyD88 deficiency on the oral innate defense, namely expression and production of several salivary antimicrobial agents. The precise association of MyD88 in the oral innate defense will require further studies to determine whether such effects actually influence susceptibility to oral infections or orally transmitted infections.

## Supporting Information

Figure S1 **qRT-PCR analysis for** ***Ltf*** **expression in SGs.** Total RNA was prepared separately from SMGs and SLGs collected from *Myd88* ^+/+^ mice and *Myd88* ^-/-^ mice at 10 weeks old (n = 8 each). Each expression level of *Ltf* was calculated relative to expression of the *Hprt1* housekeeping gene. Means of each group were shown and *P* values were calculated by unpaired Student's *t*-test. **, *P*<0.01.
(PDF)

Figure S2 **Flow cytometric analysis of B cell populations resident in SMGs.** Flow cytometry was performed on cells prepared from SMGs from four *Myd88* ^+/+^ mice and four *Myd88*-/- mice at 10 weeks old. In the dot plots, the percentage and cell number within the outlined area are shown. Data are representative of three independent experiments with three to four mice per group. A: Analysis of SMG cells obtained from four mice for expression CD45 and B220 (30,000 cells each). Another plot of each group is shown in Figure 5A. B: Analysis of SMG cells obtained from four mice for expression CD138 and B220 (30,000 cells each). Another plot of each group is shown in Figure 5B.
(PDF)

Figure S3 **Flow cytometric analysis of SMG cells stained with isotype control antibody.** Flow cytometry was performed on cells prepared from SMGs from *Myd88* ^+/+^ mice (left) and *Myd88*_-/-_ mice (right) at 10 weeks old. In the dot plots, the percentage and cell number within the outlined area are shown. Data are representative of three independent experiments. A: Analysis of SMG cells stained with FITC-labeled rat IgG2b isotype control antibody and PerCP-labeled rat IgG2a isotype control antibody (30,000 cells each). B: Analysis of SMG cells stained with PE/Cy7-labeled rat IgG2a isotype control antibody and PerCP-labeled rat IgG2a isotype control antibody (30,000 cells each). C: Analysis of SMG cells stained with PE/Cy5-labeled rat IgG2a isotype control antibody and PerCP-labeled rat IgG2a isotype control antibody (30,000 cells each). D: Analysis of SMG cells stained with PE-labeled rat IgG2a isotype control antibody, PE/Cy7-labeled rat IgG2a isotype control antibody, and PerCP-labeled rat IgG2a isotype control antibody (30,000 cells each).
(PDF)

Figure S4 **Flow cytometric analysis of B cell populations resident in spleens.** Flow cytometry was performed on cells prepared from spleens from male *Myd88* ^+/+^ mice and *Myd88* ^-/-^ mice at 10 weeks old. Data are representative of three independent experiments with four mice per group. A: Expression of CD45 in splenocytes (30,000 cells each). Black lines indicate total cells and green lines indicate B220-positive cells. The data of percentage were graphically shown as means ± SD (n = 4 per group). *, *P*<0.05 (unpaired Student's *t*-test). B: Expression of Igs in splenocytes (30,000 cells each). Black lines indicate total cells, blue lines indicate cells with low or intermediate expression of CD138 and red lines indicate cells with high expression of CD138. The data of percentage were graphically shown as means ± SD (n = 4 per group). *, *P*<0.05 (unpaired Student's *t*-test).
(PDF)

Figure S5 **Effect of MyD88 deficiency on the levels of serum basal Igs.** Sera were collected from *Myd88* ^+/+^ mice and *Myd88* ^-/-^ mice at 10 weeks old (n = 8 each) for determination of basal Ig levels by ELISA. Means of each group were shown and *P* values were calculated by unpaired Student's *t*-test. **, *P*<0.01.
(PDF)

Figure S6 **Effect of MyD88 deficiency on intestinal IgA production.** Fecal extracts were prepared from fecal pellets collected from *Myd88* ^+/+^ mice and *Myd88* ^-/-^ mice at 10 weeks old (n = 8 each) for determination of intestinal basal IgA levels by ELISA. Means of each group were shown and *P* value was calculated by unpaired Student's *t*-test.
(PDF)

Table S1 **Primer sets used in qRT-PCR analysis.**
(PDF)

Table S2 **The selected 45 genes investigated by microarray of whole SMGs.**
(PDF)
