# Supplementary material for: MyD88 Deficiency Alters Expression of Antimicrobial Factors in Mouse Salivary Glands
Source: PLoS One. 2014 Nov 21;9(11):e113333. doi: 10.1371/journal.pone.0113333 (PMC4240645; doi:10.1371/journal.pone.0113333)
Supplement: Figure S2 — Flow cytometric analysis of B cell populations resident in SMGs. Flow cytometry was performed on cells prepared from SMGs from four Myd88 +/+ mice and four Myd88-/- mice at 10 weeks old. In the dot plots, the percentage and cell number within the outlined area are shown. Data are representative of three independent experiments with three to four mice per group. A: Analysis of SMG cells obtained from four mice for expression CD45 and B220 (30,000 cells each). Another plot of each group is shown in Figure 5A. B: Analysis of SMG cells obtained from four mice for expression CD138 and B220 (30,000 cells each). Another plot of each group is shown in Figure 5B. (PDF) [file pone.0113333.s002.pdf]

Figure S2

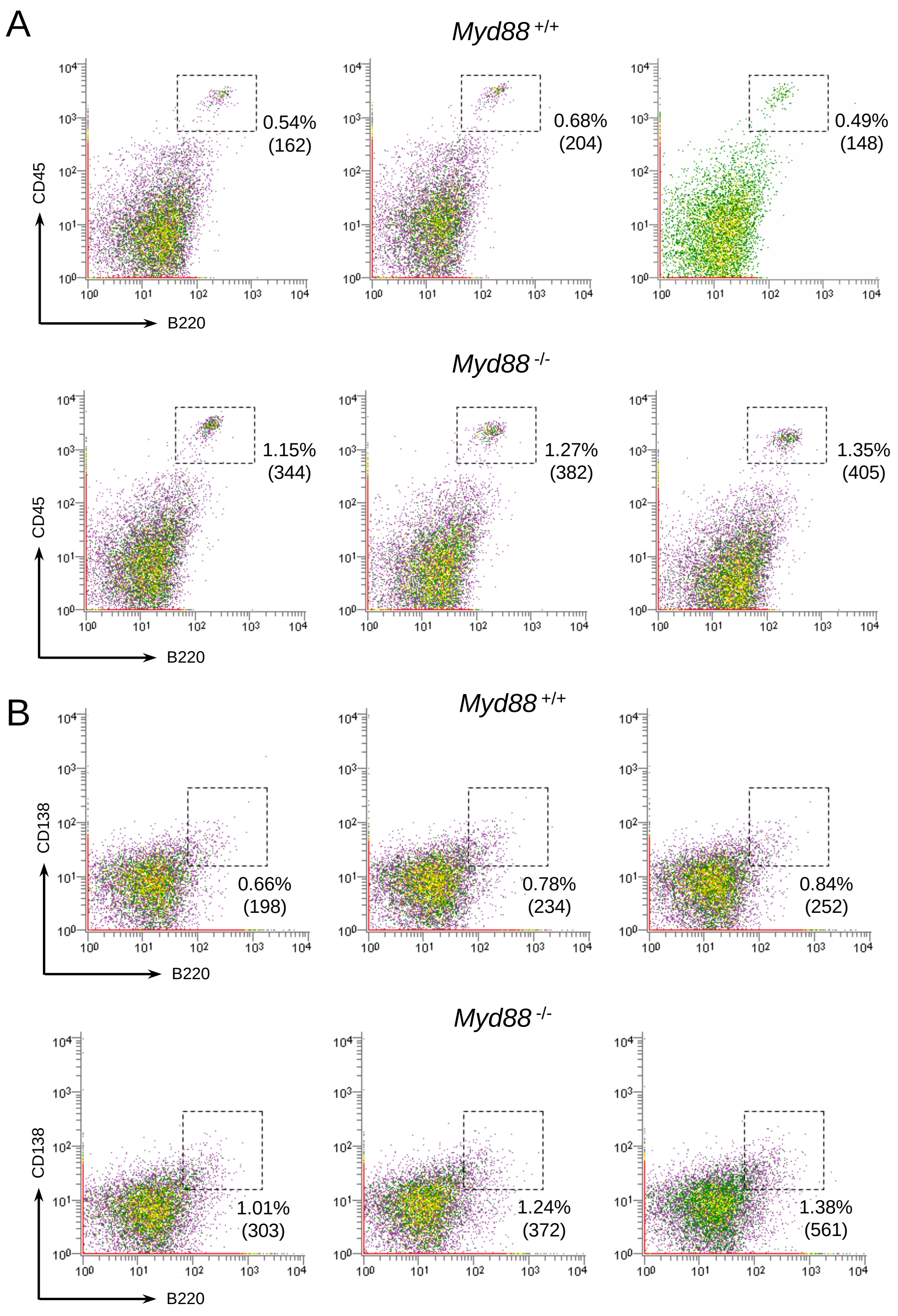

Figure S2. Flow cytometric analysis of B cell populations resident in SMGs.

Flow cytometry was performed on cells prepared from SMGs from four *Myd88*$^{+/+}$ mice and four *Myd88*$^{-/-}$ mice at 10 weeks old. In the dot plots, the percentage and cell number within the outlined area are shown. Data are representative of three independent experiments with three to four mice per group..

A: Analysis of SMG cells obtained from four mice for expression CD45 and B220 (30,000 cells each). Another plot of each group is shown in Figure 5A.

B: Analysis of SMG cells obtained from four mice for expression CD138 and B220 (30,000 cells each). Another plot of each group is shown in Figure 5B.
