# Supplementary material for: MyD88 Deficiency Alters Expression of Antimicrobial Factors in Mouse Salivary Glands
Source: PLoS One. 2014 Nov 21;9(11):e113333. doi: 10.1371/journal.pone.0113333 (PMC4240645; doi:10.1371/journal.pone.0113333)
Supplement: Figure S3 — Flow cytometric analysis of SMG cells stained with isotype control antibody. Flow cytometry was performed on cells prepared from SMGs from Myd88 +/+ mice (left) and Myd88 -/- mice (right) at 10 weeks old. In the dot plots, the percentage and cell number within the outlined area are shown. Data are representative of three independent experiments. A: Analysis of SMG cells stained with FITC-labeled rat IgG2b isotype control antibody and PerCP-labeled rat IgG2a isotype control antibody (30,000 cells each). B: Analysis of SMG cells stained with PE/Cy7-labeled rat IgG2a isotype control antibody and PerCP-labeled rat IgG2a isotype control antibody (30,000 cells each). C: Analysis of SMG cells stained with PE/Cy5-labeled rat IgG2a isotype control antibody and PerCP-labeled rat IgG2a isotype control antibody (30,000 cells each). D: Analysis of SMG cells stained with PE-labeled rat IgG2a isotype control antibody, PE/Cy7-labeled rat IgG2a isotype control antibody, and PerCP-labeled rat IgG2a isotype control antibody (30,000 cells each). (PDF) [file pone.0113333.s003.pdf]

Figure S3

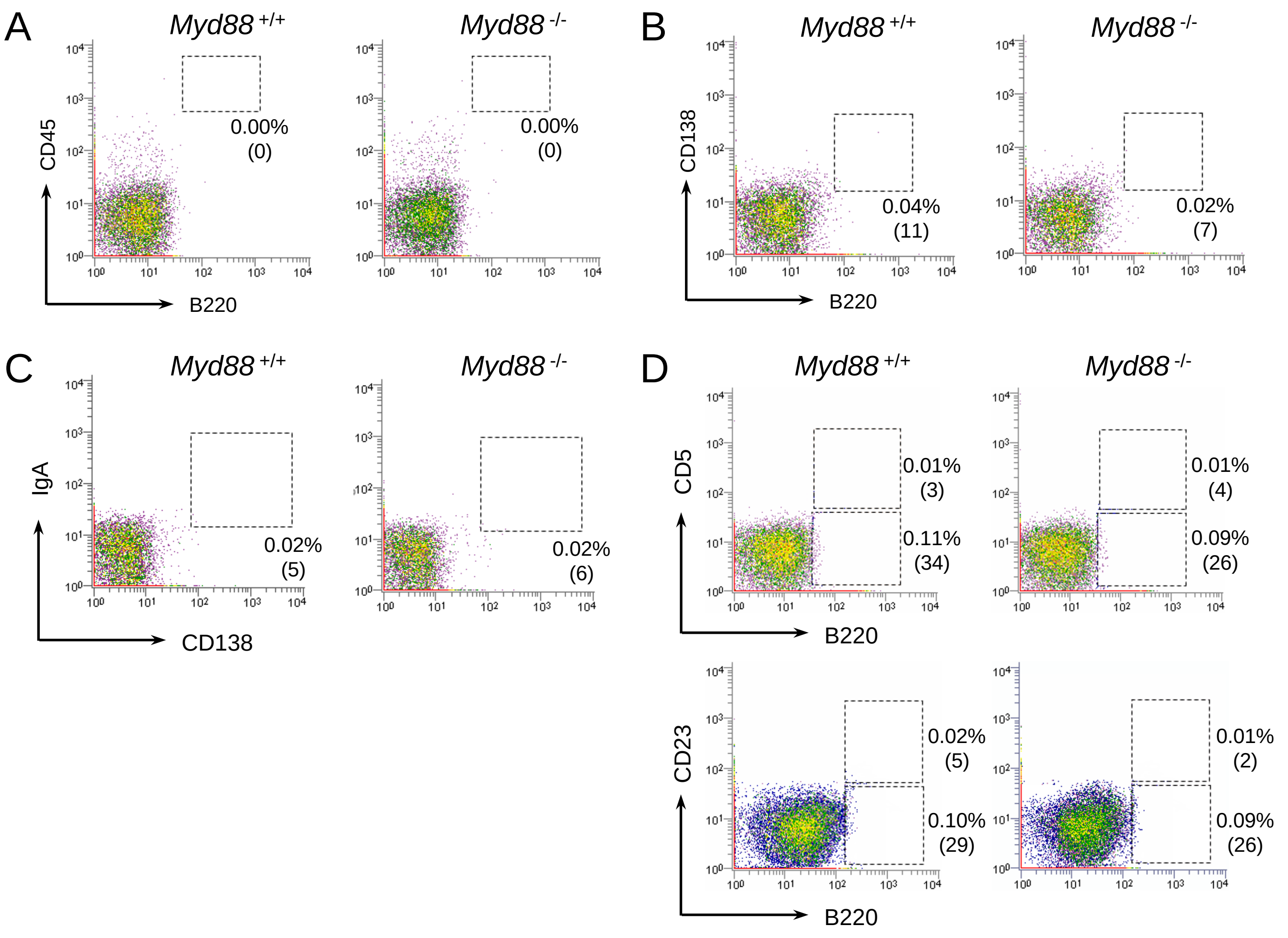

Figure S3. Flow cytometric analysis of SMG cells stained with isotype control antibody.

Flow cytometry was performed on cells prepared from SMGs from *Myd88*$^{+/+}$ mice (left) and *Myd88*$^{-/-}$ mice (right) at 10 weeks old. In the dot plots, the percentage and cell number within the outlined area are shown. Data are representative of three independent experiments.

A: Analysis of SMG cells stained with FITC-labeled rat IgG2b isotype control antibody and PerCP-labeled rat IgG2a isotype control antibody (30,000 cells each).

B: Analysis of SMG cells stained with PE/Cy7-labeled rat IgG2a isotype control antibody and PerCP-labeled rat IgG2a isotype control antibody (30,000 cells each).

C: Analysis of SMG cells stained with PE/Cy5-labeled rat IgG2a isotype control antibody and PerCP-labeled rat IgG2a isotype control antibody (30,000 cells each).

D: Analysis of SMG cells stained with PE-labeled rat IgG2a isotype control antibody, PE/Cy7-labeled rat IgG2a isotype control antibody, and PerCP-labeled rat IgG2a isotype control antibody (30,000 cells each).
