# Supplementary material for: MyD88 Deficiency Alters Expression of Antimicrobial Factors in Mouse Salivary Glands
Source: PLoS One. 2014 Nov 21;9(11):e113333. doi: 10.1371/journal.pone.0113333 (PMC4240645; doi:10.1371/journal.pone.0113333)
Supplement: Figure S4 — Flow cytometric analysis of B cell populations resident in spleens. Flow cytometry was performed on cells prepared from spleens from male Myd88 +/+ mice and Myd88 -/- mice at 10 weeks old. Data are representative of three independent experiments with four mice per group. A: Expression of CD45 in splenocytes (30,000 cells each). Black lines indicate total cells and green lines indicate B220-positive cells. The data of percentage were graphically shown as means ± SD (n = 4 per group). *, P<0.05 (unpaired Student's t-test). B: Expression of Igs in splenocytes (30,000 cells each). Black lines indicate total cells, blue lines indicate cells with low or intermediate expression of CD138 and red lines indicate cells with high expression of CD138. The data of percentage were graphically shown as means ± SD (n = 4 per group). *, P<0.05 (unpaired Student's t-test). (PDF) [file pone.0113333.s004.pdf]

Figure S4

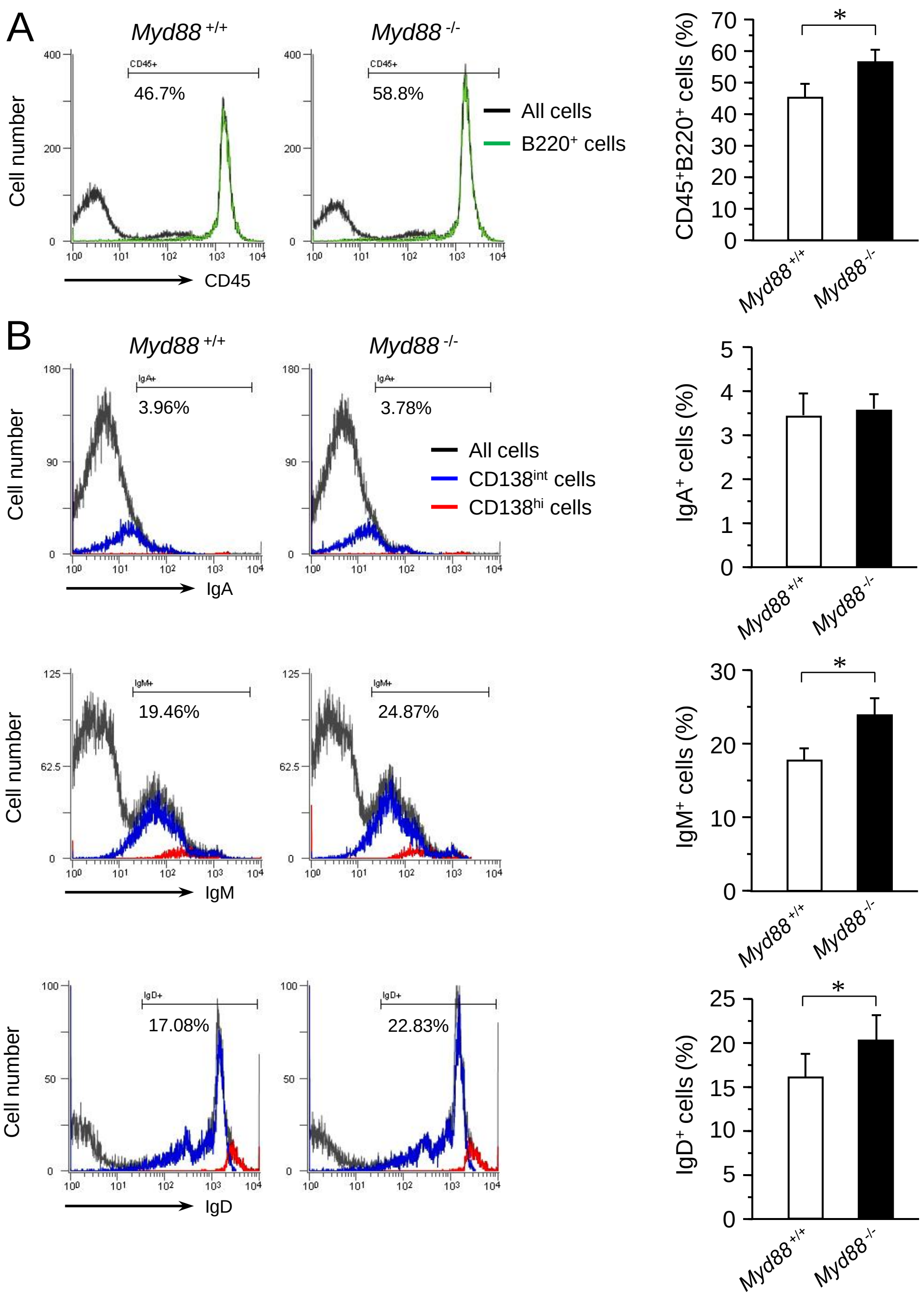

Figure S4. Flow cytometric analysis of B cell populations resident in spleens.

Flow cytometry was performed on cells prepared from spleens from male *Myd88*$^{+/+}$ mice and *Myd88*$^{-/-}$ mice at 10 weeks old. Data are representative of three independent experiments with four mice per group.

A: Expression of CD45 in splenocytes (30,000 cells each). Black lines indicate total cells and green lines indicate B220-positive cells. The data of percentage were graphically shown as means ± SD (n=4 per group). *, $P < 0.05$ (unpaired Student's *t*-test).

B: Expression of Igs in splenocytes (30,000 cells each). Black lines indicate total cells, blue lines indicate cells with low or intermediate expression of CD138 and red lines indicate cells with high expression of CD138. The data of percentage were graphically shown as means ± SD (n=4 per group). *, $P < 0.05$ (unpaired Student's *t*-test).
