# Supplementary material for: MyD88 Deficiency Alters Expression of Antimicrobial Factors in Mouse Salivary Glands
Source: PLoS One. 2014 Nov 21;9(11):e113333. doi: 10.1371/journal.pone.0113333 (PMC4240645; doi:10.1371/journal.pone.0113333)
Supplement: Figure S5 — Effect of MyD88 deficiency on the levels of serum basal Igs. Sera were collected from Myd88 +/+ mice and Myd88 -/- mice at 10 weeks old (n = 8 each) for determination of basal Ig levels by ELISA. Means of each group were shown and P values were calculated by unpaired Student's t-test. **, P<0.01. (PDF) [file pone.0113333.s005.pdf]

Figure S5

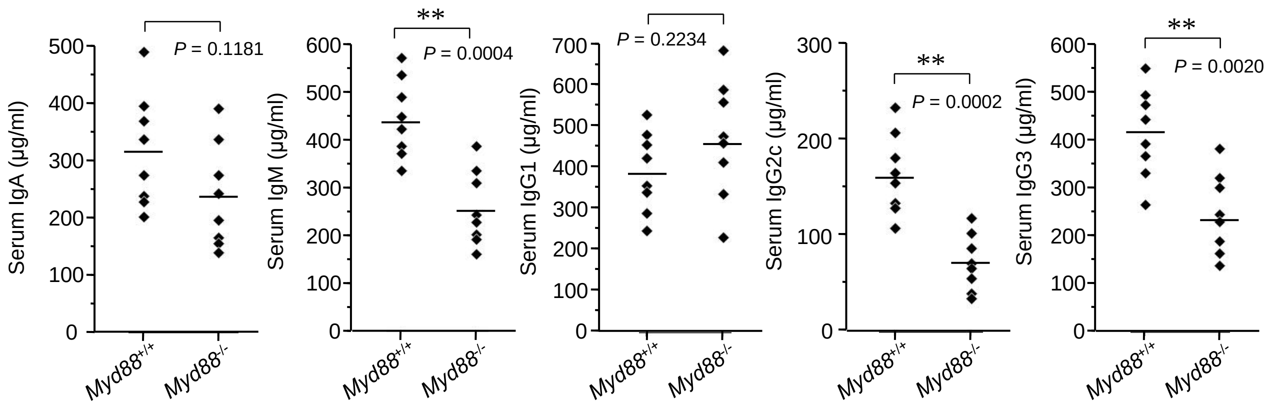

Figure S5. Effect of MyD88 deficiency on the levels of serum basal Igs.

Sera were collected from *Myd88*$^{+/+}$ mice and *Myd88*$^{-/-}$ mice at 10 weeks old (n=8 each) for determination of basal Ig levels by ELISA. Means of each group were shown and *P* values were calculated by unpaired Student's *t*-test. **, $P < 0.01$.
