# Supplementary material for: MyD88 Deficiency Alters Expression of Antimicrobial Factors in Mouse Salivary Glands
Source: PLoS One. 2014 Nov 21;9(11):e113333. doi: 10.1371/journal.pone.0113333 (PMC4240645; doi:10.1371/journal.pone.0113333)
Supplement: Table S1 — Primer sets used in qRT-PCR analysis. (PDF) [file pone.0113333.s007.pdf]

Table S1

Primer sets used in qRT-PCR analysis

Gene		Primer sequence (5’ - 3’)
Defa1	F	AGGTGACTCGCAGCCATGAA
	R	ATAGAATCAGCCTGGACCTGGAAG
Defb1	F	AGCCTCATCTGTCAGCCCAACTA
	R	TCCAAGACTTGTGAGAATGCCAAC
S100a8	Please see: RT^2 qPCR Primer Assay for Mouse *S100a8* (PPM05051F) http://www.sabiosciences.com/primerinfo.php?pcatn=PPM05051F	
S100a9	F	CCCTGACACCCTGAGCAAGA
	R	TTCTCATGACAGGCAAAGATCAAC
Ltf	F	CCTGCTTGCTAACCAGACCAGA
	R	TCACCAATACACAGGGCACAGAG
Hprt	F	TTGTTGTTGGATATGCCCTTGACTA
	R	AGGCAGATGGCCACAGGACTA
