# Supplementary material for: MyD88 Deficiency Alters Expression of Antimicrobial Factors in Mouse Salivary Glands
Source: PLoS One. 2014 Nov 21;9(11):e113333. doi: 10.1371/journal.pone.0113333 (PMC4240645; doi:10.1371/journal.pone.0113333)
Supplement: Table S2 — The selected 45 genes investigated by microarray of whole SMGs. (PDF) [file pone.0113333.s008.pdf]

Table S2

Gene Name	Description	Analogous gene in humans
Adm	adrenomedullin	*ADM*
B2m	beta-2 microglobulin	*B2M*
Bpi	bactericidal permeablility increasing protein (BPI)	*BPI*
Bpifa1	BPI fold containing family A, member 1	*PLUNC*
Bpifb1	BPI fold containing family B, member 1	*LPLUNC1*
Bpifb2	BPI fold containing family B, member 2	*BPIL1*
Calca	Calca calcitonin/calcitonin-related polypeptide, alpha	*CALCA*
Camp	cathelicidin antimicrobial peptide (LL-37)	*CAMP*
Ccl28	chemokine (C-C motif) ligand 28	*CCL28*
Csta1	cystatin A1	*CSTA*
Cstb	cystatin B	*CSTB*
Cst3	cystatin C	*CST3*
Defa1	defensin, alpha 1	*DEFA1*
Defa3	defensin, alpha 3	*DEFA3*
Defa4	defensin, alpha 4	*DEFA4*
Defb1	defensin, beta 1	*DEFB1*
Defb2	defensin, beta 2	*DEFB4A*
Defb3	defensin, beta 3	*DEFB103A*
Defb4	defensin, beta 4	*DEFB4B*
Dmbt1	deleted in malignant brain tumors 1	*DMBT1*
Fn1	fibronectin 1	*FN1*
Fgf1	fibroblast growth factor 1	*FGF1*
Fgf2	fibroblast growth factor 2	*FGF2*
Lpo	lactoperoxidase	*LPO*
Ltf	lactotransferrin	*LTF*
Lyz1	lysozyme 1	*LYZ*
Lyz2	lysozyme 2	*LYZ*
Mpo	myeloperoxidase	*MPO*
Npy	neuropeptide Y	*NPY*
Pglyrp1	peptidoglycan recognition protein 1	*PGLYRP1*
Pglyrp2	peptidoglycan recognition protein 2	*PGLYRP2*
Pglyrp3	peptidoglycan recognition protein 3	*PGLYRP3*
Pglyrp4	peptidoglycan recognition protein 1	*PGLYRP4*
Pip	prolactin induced protein	*PIP*
Prb1	proline-rich protein BstNI subfamily 1	*PRB1*
Prh1	proline rich protein HaeIII subfamily 1	*PRH1*
S100a7a	S100 calcium binding protein A7A	*S100A7*
S100a8	S100 calcium binding protein A8 (calgranulin A)	*S100A8*
S100a9	S100 calcium binding protein A9 (calgranulin B)	*S100A9*
Serpina1c	serine (or cysteine) peptidase inhibitor, clade A, member 1C	*PI3*
Sftpa1	surfactant associated protein A1	*SFTPA1*
Slpi	secretory leukocyte peptidase inhibitor	*SLPI*
Tac1	tachykinin 1 (Substance P)	*TAC1*
Trf	transferrin	*TF*
Vip	vasoactive intestinal polypeptide	*VIP*
